# Incidence and mortality of liver cancer in China in 2011

**DOI:** 10.1186/s40880-015-0056-0

**Published:** 2015-10-15

**Authors:** Ting-Ting Zuo, Rong-Shou Zheng, Si-Wei Zhang, Hong-Mei Zeng, Wan-Qing Chen

**Affiliations:** National Central Cancer Registry, National Cancer Center, Beijing, 100021 P.R. China

**Keywords:** Liver cancer, Incidence, Mortality, China

## Abstract

**Background:**

Liver cancer is a common cancer with poor prognosis in China. In this study, the national population-based cancer registration data were used to evaluate and analyze liver cancer incidence and mortality in China in 2011 and provide a reference for liver cancer prevention and control.

**Methods:**

We collected and evaluated the incidence and mortality data of liver cancer in 2011 from 177 cancer registries with qualified data. These data were used in the final analysis including calculating crude, standardized, and truncated incidences and mortalities, and estimated new liver cancer cases and deaths using age-specific rates and the corresponding populations. The national census in 2000 and Segi’s population were used for age-standardized rates.

**Results:**

The estimates of new liver cancer cases and deaths were 355,595 and 322,416, respectively, in China in 2011. The crude incidence, age-standardized rate of incidence by Chinese standard population (ASRIC), and age-standardized rate of incidence by world standard population (ASRIW) of liver cancer were 26.39/100,000, 19.48/100,000, and 19.10/100,000, respectively; the crude mortality, age-standardized rate of mortality by Chinese standard population (ASRMC), and age-standardized rate of mortality by world standard population (ASRMW) of liver cancer were 23.93/100,000,17.48/100,000, and 17.17/100,000, respectively. The incidence and mortality were higher in rural areas than in urban areas and higher in males than in females. The age-specific incidence and mortality of liver cancer increased greatly with age, particularly after 30 years and peaked at 80–84 or 85+ years.

**Conclusions:**

Liver cancer is a common cancer in China, particularly for males and residents in rural areas. Targeted prevention, early detection, and treatment programs should be carried out.

## Background

Liver cancer is one of the most common malignant tumors worldwide. According to GLOBOCAN 2012, it was estimated that 782,000 people were diagnosed with liver cancer and that 746,000 people died of this disease, accounting for 5.6% of all new cancer cases and 9.1% of all cancer deaths worldwide, making liver cancer the sixth most common type of cancer and the second leading cause of cancer-related death; moreover, more than 50% of the incident and death cases occurred in China [1]. According to the estimation of the National Central Cancer Registry (NCCR) of China, the liver cancer incidence and mortality were 27.29/100,000 and 23.76/100,000, respectively, making it the fourth most common cancer and the second leading cause of death in 2010 [2]. NCCR is responsible for cancer data collection, evaluation, and publication in China. In 2014, NCCR collected the incidence and mortality data of liver cancer for the calendar year of 2011 from 234 cancer registries. Qualified data from 177 registries were accepted to analyze the incidence and mortality status of liver cancer across the country and in different areas to provide a reference for liver cancer prevention and control.

## Data and methods

### Data resources

The incidence and death data of liver cancer in 2011 were retrieved from the National Cancer Registry Database held by the NCCR of China. The criteria for data quality were as follows: the percentage of cases morphologically verified (MV%) was higher than 66%, the percentage of death certificate-only cases (DCO%) was lower than 15%, the mortality to incidence ratio (M/I) was between 0.6 and 0.8, and the percentage of the diagnosis of unknown basis (UB%) was lower than 5%. The registries were stratified into grades A, B, C, and D according to the above criteria for data quality. Among all registries, grades A and B registries were included in the annual report, and grade C registries, with only individual indices that failed to reach the criteria of grade B, were also included as a particular reason; grade D registries were excluded. The 177 qualified registries were located in 28 provinces, autonomous regions, and municipalities directly under the central government: 77 in urban areas and 100 in rural areas. The population data came from the Bureaus of Statistics or Public Security at cancer registration areas. The 177 registries covered 175,310,169 people (88,655,668 males and 86,654,501 females) and accounted for 13.01% of the national population at the end of 2011: 98,341,507 of them in urban areas accounted for 56.10% of the population covered by the 177 registries, and 76,968,662 in rural areas accounted for 43.90%.

### National population estimates

The national population in 2011 was estimated based on the fifth National Population Census data (2000) provided by the National Statistics Bureau of China, considering the changes in the age composition, sex ratio, and proportion of urban and rural transformation released by the National Bureau of Statistics (http://data.stats.gov.cn/). The national population in 2011 was stratified by area (urban and rural), sex (male and female), and age groups (0–, 1–4, 5–84 sub-stratified by 5, and 85+ years). The changes in age-specific death probability were also adjusted when calculating the national population. Linear changes were assumed in each age group between the fifth and sixth Population Censuses.

### Statistical analyses

All liver cancer data coded as C22 in the tenth version of the International Classification of Diseases (ICD-10) were analyzed. Incident and death cases, proportions, and crude, standardized, accumulated, truncated, and age-specific incidences and mortalities were calculated using the methods recommended by the National Cancer Registration Manual [3]. The fifth Chinese National Census of 2000 and Segi’s world population were used for age-standardized rates. The above indices were calculated by region and sex stratifications. Age-specific estimates of new cases and deaths at each stratum were obtained by multiplying the age-specific incidence and mortality with the corresponding populations. The nationwide estimates of new cases and deaths were obtained by pooling the calculations [4].

## Results

### Data quality

The MV%, DCO%, M/I, and UB% of liver cancer in 2011 were 37.64%, 4.18%, 0.91, and 0.34% in the 177 registries, 39.95%, 4.08%, 0.90, and 0.46% in urban areas, and 35.10%, 4.29%, 0.92, and 0.22% in rural areas, respectively. Higher data quality was obtained in urban areas than in rural areas (Table 1).

**Table 1:** Data quality of the liver cancer registration in China in 2011

Areas	Sex	M/I	MV%	DOC%	UB%
All	Both sexes	0.91	37.64	4.18	0.34
	Males	0.91	37.43	4.08	0.34
	Females	0.92	38.24	4.45	0.35
Urban areas	Both sexes	0.90	39.95	4.08	0.46
	Males	0.89	39.74	3.95	0.43
	Females	0.93	40.58	4.44	0.55
Rural areas	Both sexes	0.92	35.10	4.29	0.22
	Males	0.92	34.86	4.22	0.25
	Females	0.91	35.76	4.45	0.13

*M/I* mortality to incidence ratio, *MV%* the percentage of cases morphologically verified, *DOC%* the percentage of death certificate-only cases, *UB%* the percentage of diagnosis of unknown basis

### Estimated liver cancer incidence

The estimated new liver cancer cases were 355,595, accounting for 10.54% of all new cancer cases and making liver cancer the third most common cancer in China in 2011. The crude incidence, age-standardized rate of incidence by Chinese standard population (ASRIC), and age-standardized rate of incidence by world standard population (ASRIW) of liver cancer were 26.39/100,000, 19.48/100,000, and 19.10/100,000, respectively. For patients aged 0–74 years, the cumulative incidence rate was 2.22%; for those aged 35–64 years, the truncated ASRIW (T-ASRIW) was 37.29/100,000. A total of 264,635 new liver cancer cases were estimated to occur in males, accounting for 13.79% of all new cancer cases and making liver cancer the third most common cancer in Chinese males in 2011. The crude incidence, ASRIC, and ASRIW in males were 38.32/100,000, 29.30/100,000, and 28.65/100,000, respectively. A total of 90,960 new cases were estimated to occur in females, accounting for 6.26% of all new cancer cases and making liver cancer the fifth most common cancer in Chinese females in 2011. The crude incidence, ASRIC, and ASRIW in females were 13.85/100,000, 9.64/100,000, and 9.55/100,000, respectively (Table 2). Males had a higher liver cancer incidence than females. The numbers of new cases in males in urban areas, rural areas, and nationwide were 3.09, 2.77, and 2.91 times greater, respectively, than those in females.

**Table 2:** Liver cancer incidence in China in 2011

Areas	Sex	No. of cases	Crude incidence (1/10^5^)	Ratio (%)	ASRIC (1/10^5^)	ASRIW (1/10^5^)	Cumulative rate (%) Age 0–74 years	T-ASRIW (1/10^5^) Age 35–64 years	Rank
All	Both sexes	355,595	26.39	10.54	19.48	19.10	2.22	37.29	4
	Males	264,635	38.32	13.79	29.30	28.65	3.30	58.22	3
	Females	90,960	13.85	6.26	9.64	9.55	1.11	15.62	5
Urban areas	Both sexes	164,528	23.82	9.11	17.07	16.75	1.93	32.01	4
	Males	124,294	35.25	12.51	26.02	25.51	2.93	50.94	3
	Females	40,234	11.90	4.96	8.11	8.00	0.92	12.27	7
Rural areas	Both sexes	191,067	29.10	12.20	22.20	21.73	2.53	43.32	4
	Males	140,341	41.51	15.17	33.03	32.18	3.71	66.62	3
	Females	50,726	15.93	7.91	11.36	11.27	1.33	19.41	5

*Ratio* the percentage of liver cancer cases in all new cancer cases, *ASRIC* age-standardized rate of incidence by Chinese standard population, *ASRIW* age-standardized rate of incidence by Segi’s world standard population, *T-ASRIW* truncated ASRIW

### Age-specific incidence

**Fig. 1:**
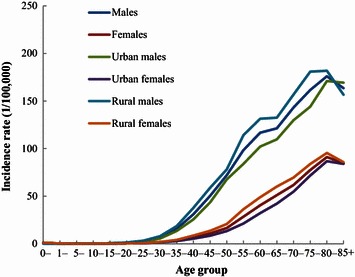
Age-specific incidence of liver cancer in China in 2011

In 2011, the age-specific incidence of liver cancer in China was low before the age of 30 years, rapidly increased after the age of 30 years, and peaked at the age of 80–84 years (Fig. 1). The age-specific incidence was remarkably higher in males than in females. Among different areas, the age-specific incidences varied with a similar curve. Notably, after the age of 30 years, the incidence in rural areas was generally higher than that in urban areas for both males and females in all age groups, except for the age group of 85+ years in males.

### Incidence difference between areas

The estimate of new liver cancer cases in urban areas in 2011 was 164,528, accounting for 9.11% of all new cancer cases and making liver cancer the fourth most common cancer in urban areas. The crude incidence, ASRIC, and ASRIW in urban areas were 23.82/100,000, 17.07/100,000, and 16.75/100,000, respectively. The estimate of new liver cancer cases in rural areas in 2011 was 191,067, accounting for 12.20% of all new cancer cases and making liver cancer the fourth most common cancer in rural areas. The crude incidence, ASRIC, and ASRIW in rural areas were 29.10/100,000, 22.20/100,000, and 21.73/100,000, respectively (Table 2). The crude incidence, ASRIC, and ASRIW in rural areas were 1.22, 1.30, and 1.29 times greater than those in urban areas, respectively (Table 2).

### Estimated liver cancer mortality

Liver cancer deaths were estimated at 322,416, accounting for 15.26% of all cancer deaths and making liver cancer the second most common cause of cancer death in China in 2011. The crude mortality, age-standardized rate of mortality by Chinese standard population (ASRMC), and age-standardized rate of mortality by world standard population (ASRMW) of liver cancer were 23.93/100,000, 17.48/100,000, and 17.17/100,000, respectively. For patients aged 0–74 years, the cumulative mortality rate was 1.98%; for those aged 35–64 years, the truncated ASRMW (T-ASRMW) was 31.64/100,000. An estimate of 239,218 liver cancer deaths occurred in males, accounting for 17.77% of all cancer deaths and making liver cancer the second most common cause of cancer death in Chinese males in 2011. The crude mortality, ASRMC, and ASRMW in males were 34.64/100,000, 26.38/100,000, and 25.90/100,000, respectively. An estimate of 83,198 liver cancer deaths occurred in females, accounting for 10.85% of all cancer deaths and making liver cancer the third most common cause of cancer death in Chinese females in 2011. The crude mortality, ASRMC, and ASRMW in females were 12.67/100,000, 8.61/100,000, and 8.51/100,000, respectively (Table 3). The mortality was higher in males than in females. The numbers of liver cancer deaths in males in urban areas, rural areas, and the whole country were 2.96, 2.80, and 2.87 times greater, respectively, than those in females.

**Table 3:** Liver cancer mortality in China in 2011

Areas	Sex	No. of cases	Crude mortality (1/10^5^)	Ratio (%)	ASRMC (1/10^5^)	ASRMW (1/10^5^)	Cumulative rate(%) Age 0–74 years	T-ASRMW (1/10^5^) Age 35–64 years	Rank
All	Both sexes	322,416	23.93	15.26	17.48	17.17	1.98	31.64	2
	Males	239,218	34.64	17.77	26.38	25.90	2.97	49.81	2
	Females	83,198	12.67	10.85	8.61	8.51	0.97	12.83	3
Urban areas	Both sexes	146,618	21.22	13.75	15.04	14.79	1.68	26.51	2
	Males	109,610	31.09	16.33	22.86	22.51	2.57	42.53	2
	Females	37,008	10.94	9.36	7.26	7.15	0.80	9.82	4
Rural areas	Both sexes	175,798	26.78	16.80	20.23	19.83	2.30	37.50	2
	Males	129,608	38.34	19.20	30.37	29.70	3.41	58.24	2
	Females	46,190	14.50	12.43	10.13	10.02	1.16	16.22	3

*Ratio* the percentage of liver cancer cases in all cancer deaths, *ASRMC* age-standardized rate of mortality by Chinese standard population, *ASRMW* age-standardized rate of mortality by Segi’s world standard population, *T-ASRMW* truncated ASRMW

### Age-specific mortality

**Fig. 2:**
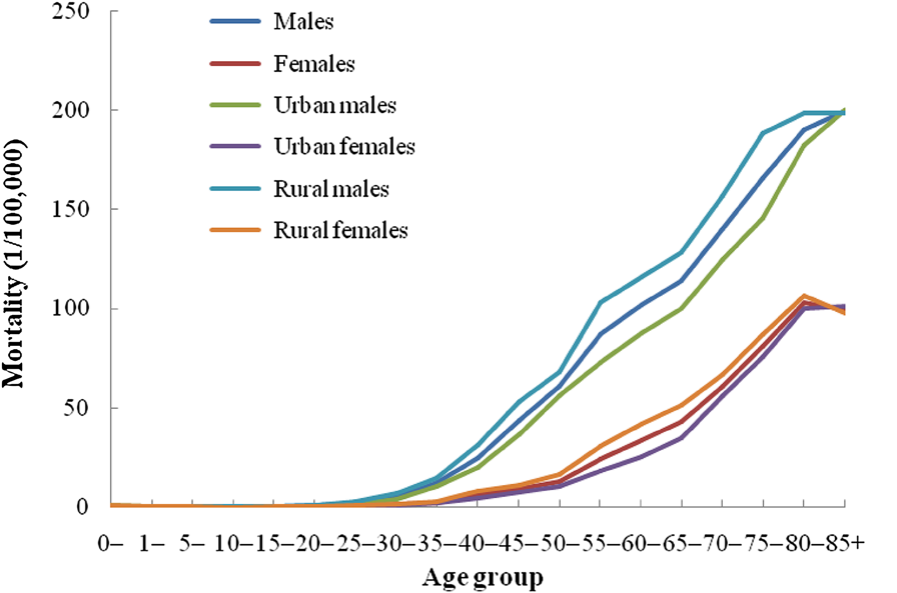
Age-specific mortality of liver cancer in China in 2011

In 2011, the age-specific mortality of liver cancer in China was similar to its age-specific incidence; it was low before the age of 30 years, rapidly increased after the age of 30 years, and peaked at the age of 80–84 or 85+ years (Fig. 2). The age-specific mortality was higher in males than in females. Among different areas, the age-specific mortalities were similar, only with a slight difference in the peak age; after the age of 30 years, the mortality in rural areas was generally higher than that in urban areas for both males and females in all age groups, except for the age group of 85+ years.

### Mortality difference between areas

Liver cancer deaths in urban areas were estimated at 146,618, accounting for 13.75% of all cancer deaths and making liver cancer the second most common cause of cancer death in urban areas in 2011. The crude mortality, ASRMC, and ASRMW in urban areas were 21.22/100,000, 15.04/100,000, and 14.79/100,000, respectively. Liver cancer deaths in rural areas were estimated at 175,798, accounting for 16.80% of all cancer deaths and making liver cancer the second most common cause of cancer death in rural areas in 2011. The crude mortality, ASRMC, and ASRMW in rural areas were 26.78/100,000, 20.23/100,000, and 19.83/100,000, respectively (Table 3). The mortality was higher in rural areas than in urban areas. The crude mortality, ASRMC, and ASRMW in rural areas were 1.26, 1.34, and 1.34 times greater than those in urban areas, respectively.

## Discussion

Liver cancer is a serious problem in developing regions, where 83% of new cancer cases worldwide occurred in 2012. According to GLOBOCAN 2012, the ASRIW and ASRMW of liver cancer in 2012 were 10.1/100,000 and 9.5/100,000 worldwide, 12.0/100,000 and 11.5/100,000 in developing countries, and 5.4/100,000 and 4.6/100,000 in developed countries, respectively [5]. In the present study, there were 355,595 new liver cancer cases and 322,416 deaths that occurred in 2011, making liver cancer the fourth most common cancer and the second leading cause of cancer-related death in China. The ASRIW and ASRMW of liver cancer in China in 2011 were 19.10/100,000 and 17.17/100,000, respectively; compared with the data of GLOBOCAN in 2012, both the incidence and mortality of liver cancer in China were at high levels worldwide. However, in China, compared with the liver cancer data in 2010 [6], the crude incidence, ASRIC, and ASRIW in 2011 were lower; the crude mortality in 2011 was similar to that in 2010, and ASRMC and ASRMW in 2011 were lower.

In China, in 2011, both the incidence and mortality of liver cancer in males were significantly higher than those in females. The reasons for the higher rates in males may be related to genetic factors [7, 8] and their high proportion of alcohol consumption, which is a risk factor of liver cancer [9, 10]. Both the incidence and mortality of liver cancer were higher in rural areas than in urban areas. The higher incidence in rural areas is most likely related to their easy access to risk factors, such as aflatoxin exposure, unhealthy diet habits, polluted water, and blood transfusion [11, 12]. Due to the low level of medical treatment and limited medical resources in rural areas, the mortality in rural areas was higher than that in urban areas. From a population-based cancer survival study, the 5-year relative survival rate of liver cancer patients was 10.1% in China, and the survival rate of rural patients was less than half of that of their urban counterparts [13]. The government should enhance the quality of health services, improve the rural medical service level, and balance medical resources between urban and rural areas to bridge the gap. The survival rate of patients with liver cancer was generally low in both developed and developing regions. According to the report from a global surveillance of cancer survival, the 5-year survival rate of liver cancer patients is below 20% everywhere in Europe, in the range of 15%–19% in North America, and as low as 7%–9% in Mongolia and Thailand [14]. The prognosis for patients with liver cancer is very poor, and advances in cancer prevention, early detection, and treatment are the main means to reduce the mortality of liver cancer.

With changes in dietary habits and the implementation of neonatal hepatitis B virus (HBV) vaccination for years, infections by aflatoxin and HBV have been effectively controlled [15, 16]. The trend analyzed from Qidong, Jiangsu showed that the age-standardized rate is decreasing, whereas the crude incidence of liver cancer is still increasing mainly because of population aging [17]. To decrease the incidence and mortality in males and in rural areas, targeted prevention, early detection, and treatment programs should be carried out, enhancing secondary prevention based on strengthening primary prevention.
